# Functional Mimetics of the HIV-1 CCR5 Co-Receptor Displayed on the Surface of Magnetic Liposomes

**DOI:** 10.1371/journal.pone.0144043

**Published:** 2015-12-02

**Authors:** Alona Kuzmina, Karin Vaknin, Garik Gdalevsky, Maria Vyazmensky, Robert S. Marks, Ran Taube, Stanislav Engel

**Affiliations:** 1 The Shraga Segal Department of Microbiology and Immunology, Faculty of Health Sciences, Ben-Gurion University of the Negev, Beer-Sheva, Israel; 2 Department of Clinical Biochemistry and Pharmacology, Faculty of Health Sciences, Ben-Gurion University of the Negev, Beer-Sheva, Israel; 3 National Institute for Biotechnology in the Negev, Beer-Sheva, Israel; 4 The Department of Biotechnology Engineering, Faculty of Engineering, Ben-Gurion University of the Negev, Beer-Sheva, Israel; Deutsches Primatenzentrum GmbH - Leibniz-Institut fur Primatenforschung, GERMANY

## Abstract

Chemokine G protein coupled receptors, principally CCR5 or CXCR4, function as co-receptors for HIV-1 entry into CD4^+^ T cells. Initial binding of the viral envelope glycoprotein (Env) gp120 subunit to the host CD4 receptor induces a cascade of structural conformational changes that lead to the formation of a high-affinity co-receptor-binding site on gp120. Interaction between gp120 and the co-receptor leads to the exposure of epitopes on the viral gp41 that mediates fusion between viral and cell membranes. Soluble CD4 (sCD4) mimetics can act as an activation-based inhibitor of HIV-1 entry *in vitro*, as it induces similar structural changes in gp120, leading to increased virus infectivity in the short term but to virus Env inactivation in the long term. Despite promising clinical implications, sCD4 displays low efficiency *in vivo*, and in multiple HIV strains, it does not inhibit viral infection. This has been attributed to the slow kinetics of the sCD4-induced HIV Env inactivation and to the failure to obtain sufficient sCD4 mimetic levels in the serum. Here we present uniquely structured CCR5 co-receptor mimetics. We hypothesized that such mimetics will enhance sCD4-induced HIV Env inactivation and inhibition of HIV entry. Co-receptor mimetics were derived from CCR5 gp120-binding epitopes and functionalized with a palmitoyl group, which mediated their display on the surface of lipid-coated magnetic beads. CCR5-peptidoliposome mimetics bound to soluble gp120 and inhibited HIV-1 infectivity in a sCD4-dependent manner. We concluded that CCR5-peptidoliposomes increase the efficiency of sCD4 to inhibit HIV infection by acting as bait for sCD4-primed virus, catalyzing the premature discharge of its fusion potential.

## Introduction

G protein coupled receptors (GPCRs), fundamental players in virtually all physiological processes, are causatively involved in many pathological states [1]. Key players in HIV infection, the chemokine GPCRs CCR5 and CXCR4 function as co-receptors for viral entry into CD4^+^ target cells [2]. HIV infection is initiated *via* binding of the viral envelope glycoprotein (Env), a homotrimer whose protomers comprise two subunits each (gp120 and gp41 glycoproteins), to the host CD4 receptor on the surface of CD4^+^ T lymphocytes. Env binding to CD4 induces a cascade of conformational changes in the former that culminate in *i*) exposure of a high-affinity co-receptor binding epitope on gp120 and *ii*) formation of a gp41 pre-hairpin intermediate that facilitates the virus-cell fusion process [3]. Overall, the viral gp120-gp41 trimer transitions from an unconjugated, high-potential-energy state to a bound, low-potential-energy state that promotes viral-cell membrane fusion [4]. In its inactive state, the gp120-gp41 complex allows HIV-1 to “hide” its Env epitopes until engagement with its target cell, thus providing an efficient steric mechanism that enables the Env epitopes to escape immune recognition by potentially neutralizing antibodies.

Attempts to inhibit HIV entry have utilized CD4 mimetics in the form of soluble CD4 (sCD4) or small molecules [2]. Similar to native CD4, these molecules were shown to induce conformational changes in the viral glycoproteins [5, 6]. However, in contrast to the cell-membrane expressed CD4, sCD4 exhibits opposing effects on HIV-1 infectivity, such that it can either enhance or inhibit viral infectivity, depending on the mimetic concentration and on the viral isolate [2, 7]. While the inhibition mechanism of CD4 mimetics remains unclear, the lack of correlation between sCD4 inhibition potency and its affinity to soluble gp120 indicates that inhibition does not rely solely on competition [2, 8]. Some soluble CD4 mimetics, including sCD4 or small-molecule compounds, were shown to prematurely prime Env into an active but metastable conformational state that consequently decayed, leading to an irreversible loss of Env membrane-fusion potential and subsequent inhibition of virus infectivity [2, 5, 6, 9]. The life span of the metastable Env complex intermediate varies for different Env HIV isolates independently of gp120 affinity for CD4, implicating metastable state inactivation kinetics in defining the susceptibility of HIV-1 isolates to inhibition by sCD4 [9]. The overall lack of sCD4 efficacy in reducing viral loads in HIV infected individuals may thus signify a more general phenomenon in which the kinetics of sCD4-induced HIV-1 inactivation are too slow to inhibit viral infectivity before virus fusion to target cells. Therefore, to efficiently exploit the inhibitory potential of sCD4 (or its small-molecule analogs), research should focus on developing pharmacological tools to accelerate the inactivation kinetics of the metastable Env intermediate.

We hypothesized that the transition from a metastable, active intermediate to an inactive state may be accelerated by the exposure of sCD4-primed viruses to a functional mimetic of HIV-1 co-receptors. Early infection of HIV is primarily dominated by R5-tropic viruses (use CCR5 as co-receptor), which infect macrophages and primary T cells. The presence of R5 HIV-1 isolates early after seroconversion indicates that they play an important role in initiating infection [10, 11]. Therefore, we constructed structural mimetics of the entire gp120-recognition epitope of the CCR5 co-receptor. These comprised peptides derived from the N-terminus and the second extracellular loop (ECL2) functionalized with a hydrophobic anchor to allow the peptides to be displayed on the surface of lipid-coated magnetic beads. Our study demonstrates that CCR5-peptidoliposomes efficiently interact with a soluble recombinant gp120 and, importantly, that they enhance the ability of sCD4 to specifically inhibit R5-tropic HIV infection.

## Materials and Methods

### Preparation of magnetic peptidoliposomes

The synthetic lipids were obtained as chloroform solutions from Avanti Polar Lipids (Alabaster, AL, USA). Magnetic liposomes were prepared according to the procedure described by Mirzabekov et.al. [12] with modifications. A total of 10 mg of chloroform-dissolved lipids (1-palmitoyl-2-oleoyl-*sn*-glycero-3-phosphocholine (POPC), 1-palmitoyl-2-oleoyl-*sn*-glycero-3-phosphoethanolamine (POPE) and dimyristoylphosphatidic acid (DMPA)), mixed in a molar ratio of 6:3:1, were dried under vacuum. One milliliter of phosphate buffered saline (PBS) was added and a mixed lipid suspension was obtained by ultrasonication for 10 min at room temperature (r.t.) in a bath sonicator (ELMA S-30H, Singen, Germany). Suspensions of the head-group-modified lipids 1,2-dioleyl-*sn*-glycero-3-phosphoethanolamine-n-(biotinyl) (Biotinyl-DOPE) and dioleoylphosphoethanolamine-lissamine rhodamine B (Rhodamine-DOPE) (1 mg/ml) were prepared separately using the same protocol. All lipid suspensions were kept at −80°C until use.

To prepare magnetic liposomes, approximately 1.7 × 10^8^ streptavidin-coated beads (1 μm silica-coated super-paramagnetic beads, BcMag, BioClone Inc., San Diego, CA) were washed twice with PBS and combined with 1 mg lipids in the aforementioned lipid suspension and 10 μg lipids (1%) in a Biotinyl-DOPE suspension. The mixture was dissolved in 1 ml of solubilization buffer S (20 mM Tris-HCl, pH 7.5, 100 mM (NH_4_)_2_SO_4_, 10% (v/v) glycerol and 1% (w/v) CHAPSO) and incubated for 1 h at 4°C. After incubation, the detergent was slowly removed by dialysis for 24 h at 4°C against 20 mM Tris-HCl, pH 7.5, 100 mM (NH_4_)_2_SO_4_ and 10% (v/v) glycerol buffer, using a 10 kDa cut-off membrane (Slide-A-Lyzer 10 K; Pierce, Rockford, IL). The resulting magnetic liposomes were washed twice with PBS and stored in PBS at 4°C until use.

Analysis of the lipid layer formation was performed by monitoring the level of incorporation of a fluorescently labeled lipid (Rhodamine-DOPE) into the magnetic liposomes [12]. The magnetic liposomes were prepared as described above in the presence of 1% (by weight) Rhodamine-DOPE. The bead membranes were dissolved in the solubilization buffer, and rhodamine absorbance was measured at 550 nm in the resolubilized lipids. The total amount of lipids in the sample was estimated using a calibration curve of rhodamine in the same lipid mixture. The homogeneity of the rhodamine-labeled liposomal population was analyzed by a FACS Calibur II instrument (BD) equipped with an Argon 488 nm laser and a 525/50 band pass filter. Acquisition and analysis were done using Cellquest software V5.1.1 (BD).

To prepare magnetic peptidoliposomes, palmitoyl-functionalized peptides were added (at the peptide/lipid molar ratio indicated in the text) as a 1-mg/ml peptide solution (either in 70% ethanol or buffer S) to 1 mg of lipids in the mixed lipid suspension (described above) prior to solubilization by buffer S, and the magnetic liposomes were prepared as described above. The modified palmitoyl-functionalized peptides were synthesized by ChinaPeptides Co., Ltd (Shanghai, China). Peptide identities and purities (> 90%) were confirmed by mass spectrum and high performance liquid chromatography analyses.

### Assessment of peptide incorporation into peptidoliposomes

To assess the extent of peptide incorporation into the bilayer and its accessibility to the solvent, approximately 1 × 10^7^ peptidoliposomes were suspended in 0.5 ml 1% bovine serum albumin blocking solution (KPL, Gaithersburg MD, USA) and incubated for 1 h at r.t. in the presence of 2.5 μg of a primary antibody against the peptide and 2.5 μg of a secondary horseradish-peroxidase-conjugated antibody. The peptidoliposomes were washed three times with 500 μl of a washing solution (KPL, Gaithersburg MD, USA) and once with 500 μl of PBS. Peroxidase activity was detected with the SureBlue TMB (tetramethylbenzidine) peroxide substrate (KPL, USA) at 450 nm.

### Interaction of peptidoliposomes with soluble recombinant gp120

His-tagged recombinant gp120 was kindly provided by Dr. Jacob Anglister (Weizmann Institute of Science, Rehovot, Israel). The protein, a monomeric gp120 core derived from the JRFL strain of HIV-1, contains V3 and lacks the V1/V2 loop region. Four mutations in the first α-helix (located on the non-neutralizing face of the viral spike) were introduced into the protein to promote monomerization [13, 14].

Peptidoliposomes were incubated for 1 h at 37°C in the presence of 4 μM soluble gp120 followed by incubation for 60 min in the presence of HRP-conjugated Anti-6His antibody. Peroxidase activity was determined as described above. When the M48 peptide (GL Biochem, China), a soluble CD4 mimetic (sCD4M48) [15], was used in the experiments, it was preincubated in 50 mM sodium phosphate buffer, pH 7.8, containing 5 mM reduced and 0.5 mM oxidized glutathione, for 30 min at r.t. to induce the formation of intramolecular disulfide bonds [16].

### Inhibition of HIV-1 infectivity by peptidoliposomes

HIV-1 recombinant lentiviruses (JRFL- and ADA-pseudotyped R5-tropic and HXB2-pseudotyped X4-tropic) expressing the HIV-Tat protein and the blue fluorescent protein (BFP) reporter driven by the LTR HIV promoter were prepared as described earlier [17]. Briefly, single-round particles were produced by calcium phosphate co-transfection of HEK-293T cells with the following plasmids: Lentiviral transfer vectors expressing the HIV-Tat protein positioned in *cis* to a BFP reporter gene (LTR-Tat-BFP), the HIV structural and accessory gene (HIV gag/pol), pRev, pTat and the indicated HIV envelope (JRFL, ADA or HXB2). Cell supernatant was harvested, centrifuged and filtered through a 0.45-μm filter 48 h post transfection. Aliquots of viral stocks were frozen at −80°C. Viral titers were determined by transduction of HEK-293T cells using serial viral dilutions, and 48 h post transduction, cells were harvested and analyzed for their BFP expression by FACS analysis.

The virus (5×10^4^ infectious viral particles) was mixed with peptidoliposomes (5×10^5^ beads) in 100 μl PBS and incubated for 30 min in an orbital shaker (150 rpm) at 37°C. The suspension was then gently centrifuged at 1,000×g for 90 min with a brief mixing every 30 min. After 2 h of incubation (total), the virus was separated from the peptidoliposomes using a magnetic field, and 20 μl of virus supernatant was mixed with 180 μl of serum-free Dulbecco's modified Eagle's medium (DMEM). Viral infection of TZM-HeLa-β-Gal (CCR5^+^/CXCR4^+^) containing an integrated HIV-LTR-β-gal reporter gene was performed for 4 h at 37 °C in a whole range of viral dilutions until reaching 0% of infection. This ensures that infectivity and inhibition effects of the mimetics are measured at their linear range. Infection media were then removed from cells, which underwent a washing step with PBS followed by supplementation with complete growth media. Cells were subjected to β-galactosidase staining 48 h post infection according to the manufacturer’s instructions (Promega). The viral supernatant and the peptidoliposomes (twice-washed with PBS) were analyzed by p24 enzyme-linked immunosorbent assay (ELISA) for the presence of HIV-p24 antigen.

All data are shown as mean ± S.E.M. and are representative of at least three independent experiments each performed in triplicate. Statistical analysis entailed comparisons of two groups of data by unpaired parametric two-tailed *t*-tests using Prism 6 software (GraphPad), and *p* values less than 0.05 were considered significant and indicated with an asterisk (*) in the figures.

## Results

R5-tropic CD4-activated HIV-1 recognizes two major epitopes of the CCR5 ectodomain: the N-terminus and the second extracellular loop (ECL2) [18–21]. CD4 binding to gp120 results in the exposure of the third (variable among HIV-1 strains) gp120 loop (V3), which extends from the gp120 core toward the co-receptor [22]. The conserved β-turn at the tip of V3 interacts with the ECL2, while the stem and base of V3, including the elements of the conserved, conformationally flexible bridging sheet, interact with the N-terminus [2, 20, 23, 24].

Modified peptides corresponding to the N-terminus and ECL2 of human CCR5 were synthesized to contain a hydrophobic moiety (palmitoyl group), which facilitates spontaneous peptide incorporation in and display on the artificial hydrophobic matrix (lipid bilayer) deposited on the surface of magnetic beads to yield magnetic CCR5-peptidoliposomes. Peptide embedment in an artificial lipid membrane imposes spatial constraints on the functionalized peptides similar to those present in native receptors while preserving the peptides’ ability to translate unrestrictedly along the membrane plane. We hypothesized that such an arrangement would facilitate the reconstruction of a composite gp120-binding epitope that accurately reflects the epitope’s 3D complexity to provide better CCR5 functional mimicry compared to that possible with soluble CCR5-derived peptides.

### Construction of magnetic liposomes

**Fig 1:**
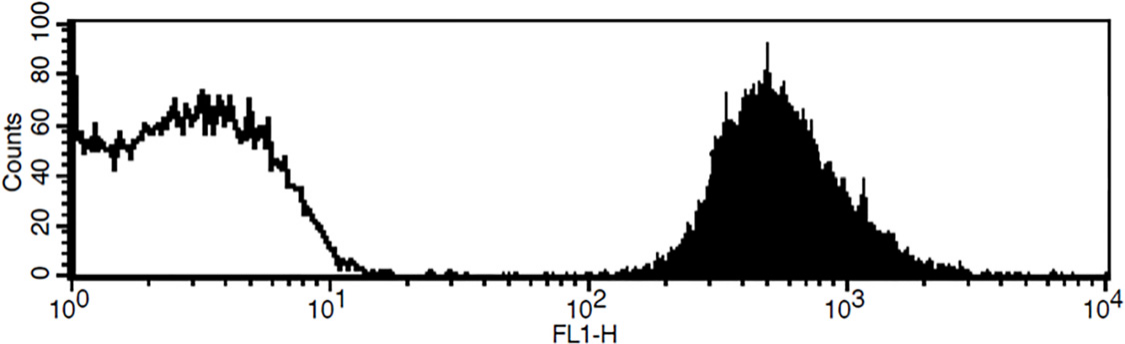
Magnetic liposome population is homogeneous. The magnetic liposomes were prepared using a lipid mixture POPC:POPE:DMPA (molar ratio 6:3:1) containing 1% Biotinyl-DOPE in the presence (black peak) or absence (white peak) of 1% Rhodamine–DOPE, and analyzed by FACS as described in the Materials and Methods. FL1-H designates the height of the photon peak obtained by using a 525/50 band pass filter (FL1). The figure shows the data for an experiment representative of two similar experiments.

Magnetic liposomes were formed by incubating streptavidin-coated magnetic beads with a mixture of detergent-solubilized lipids in the presence of 1% Biotinyl-DOPE that stabilizes the resulting lipid bilayer and prevents shedding [12]. The lipid content of the resulting magnetic liposomes, determined using the fluorescent tracer rhodamine-DOPE, was ~ 200 μg per 10^8^ beads, which is higher than a theoretical value (∼110 μg) calculated for a lipid bilayer on the surface of 1.0-μm beads using the formula m = 2∙S∙n∙M/ρ∙N_A_, where *m* is the approximate total mass of lipids, *M* is the average MW of the lipids (740 g∙mol^-1^), *n* is the number of beads, *S* is the estimated effective surface area of the bead, *ρ* is the approximate area occupied by a single lipid molecule (60 Å^2^), and *N*_*A*_ is Avogadro’s number. The discrepancy between the theoretical and experimental values for bead lipid content may be explained, at least partially, by bead surface irregularities [12]. Fluorescence-activated cell sorting (FACS) analysis of the rhodamine-labeled magnetic liposomes revealed a narrow peak of fluorescence, indicating a high degree of homogeneity within the labeled liposomal population (Fig 1). Using a similar protocol for the magnetic liposome formation, it has previously been shown by confocal microscopy that no lipid vesicles or other structures (>0.1 μm) were present on the surfaces of the resulting liposomes, a result that is in agreement with the notion that the magnetic beads are surrounded by a single lipid bilayer with rather small irregularities [12].

### Construction of CCR5-peptidoliposomes

The following palmitoylated peptidomimetics of the CCR5 gp120-recognition epitopes were synthesized:

(1) NT-2Y-CCR5-PAL:
  MDYQVSSPIY-(SO_3_H)DINY-(SO_3_H)YTSEPAQKINVKQ-(Lys-PAL)-NH_2_.
  NT-2Y-CCR5-PAL corresponds to the first 27 amino acids of the CCR5 N-terminus. The peptidomimetic was functionalized at its C-terminus (adjacent to the membrane) with a palmitoyl group (PAL) via the side-chain of a lysine. The CCR5 N-terminus is acidic and contains a number of sulfo-tyrosine residues [14, 23, 25–27]. The sulfo-tyrosine residues are thought to bind to a highly conserved pocket at the base of gp120 V3 and the C4 region, resulting in the stabilization of the V3 stem [20]. Thus, the N-terminus peptidomimetic was synthesized to contain sulfo-tyrosine residues (Y-(SO_3_H)) at positions 10 and 14.
(2) ECL2-CCR5-2PAL:
  (PAL)-TRSQKEGLHYTCSSHFPYSQYQFWKNFQTL-(Lys-PAL)-NH_2_.
  ECL2-CCR5-2PAL corresponds to the CCR5 residues 167–197 constituting the ECL2. To furnish this peptidomimetic with the spatial constraints characteristic of native receptors, the peptide was functionalized with a PAL group at both the N- and C-termini to enable them to anchor simultaneously to the lipid bilayer. Cys-178 at the middle of the CCR5 ECL2 is known to form a conserved disulfide bond connecting the ECL2 to the extracellular end of the transmembrane helix 3 (TM3) in most class A GPCRs. Although important for cytokine binding, this disulfide bond is not required for the CCR5 co-receptor activity that mediates HIV-1 entry [28].

**Fig 2:**
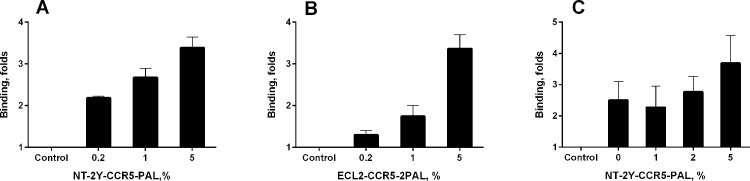
Palmitoylated CCR5-peptidomimetics can be displayed on liposome surface. Peptidoliposomes were generated in the presence of increasing concentrations (molar %) of NT-2Y-CCR5-PAL (A) or ECL2-CCR5-2PAL (B) in the lipid mixture. (C) Peptidoliposomes were formed in the presence of 1% ECL2-CCR5-2PAL and the indicated concentrations of NT-2Y-CCR5-PAL in the lipid mixture. Peptidoliposomes were reacted with anti-CCR5 N-terminus polyclonal antibody (ab 7346, Abcam) (A) or with anti-CCR5 ECL2 (raised against a synthetic peptide 2D7-2SK [44]) polyclonal antibody (ab 36818, Abcam) (B and C), followed by a secondary HRP-conjugated antibody (ab 7090, Abcam) and analyzed as described in the Materials and Methods. Control – peptide-free magnetic liposomes. The data are mean ± S.E.M. calculated from three independent experiments each performed in triplicate.

The palmitoylated peptidomimetics were mixed with the lipids in varied proportions during magnetic liposome preparation, and the efficiency of modified peptide incorporation into the lipid bilayer was assessed using specific antibodies recognizing the N-terminus and ECL2 CCR5 epitopes (Fig 2A and 2B). Upon incorporation, the peptidomimetics appeared to orient correctly, such that they were facing the solvent and accessible for interaction with soluble agents such as antibodies. Since the ultimate goal is to create liposomes that contain a combination of peptidomimetics, we tested the effect of the presence of increasing concentrations of one modified peptide on the incorporation efficiency of the other. No adverse effect on the incorporation efficiency of ECL2-CCR5-2PAL was observed when the concentration of NT-2Y-CCR5-PAL was increased in the lipid mixture (Fig 2C).

**Fig 3:**
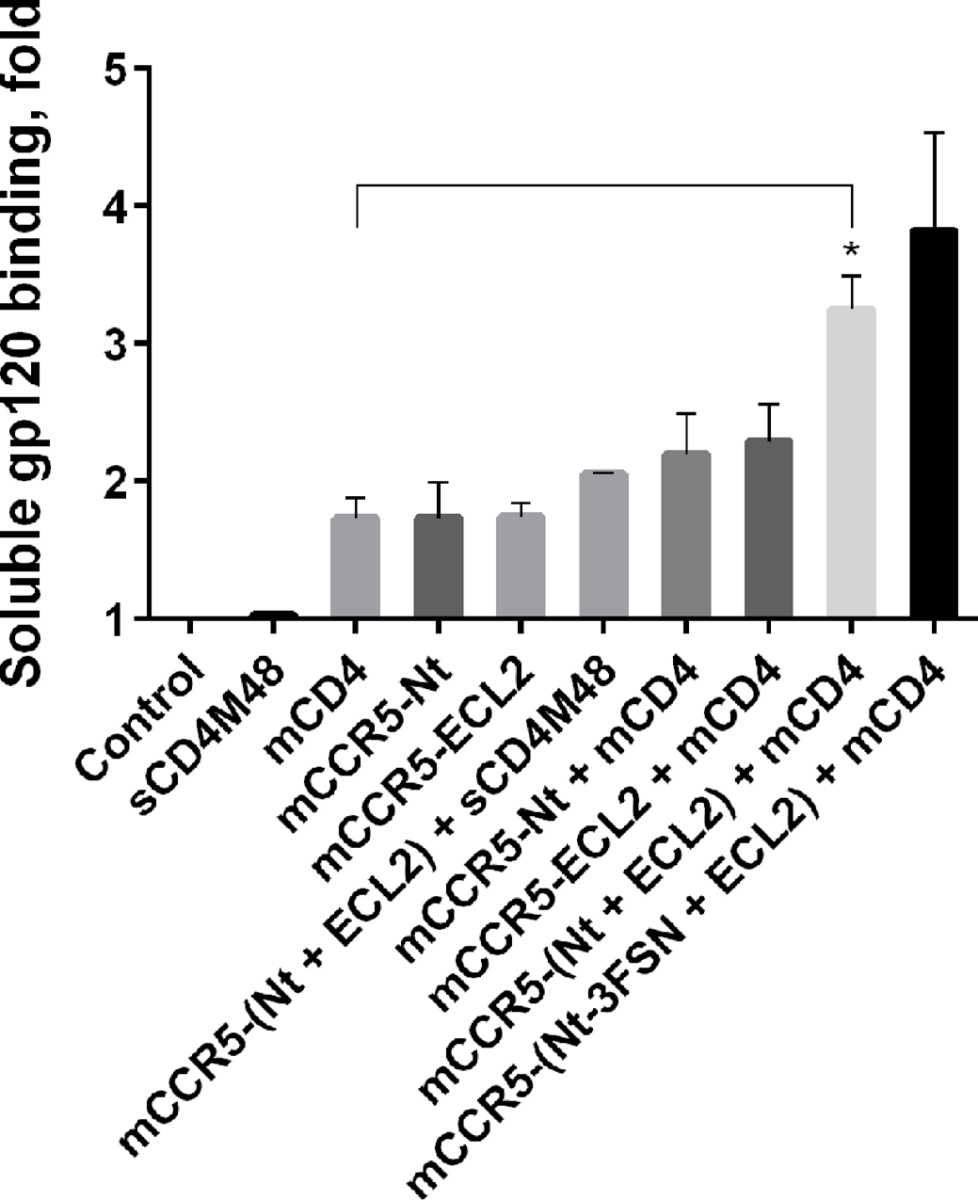
CCR5-peptidoliposomes bind soluble recombinant gp120. Peptidoliposomes were incubated with 4 μM soluble recombinant His-tagged gp120 for 1 h at 37°C and the binding was assessed as described in the Materials and Methods. Mimetics used: mCD4 –CD4M48-PAL (1%); mCCR5-Nt – NT-2Y-CCR5-PAL (1%); mCCR5-ECL2 –ECL2-CCR5-2PAL (1%); mCCR5-Nt-3FSN–NT-3FSN-CCR5-PAL (1%); sCD4M48 –soluble M48 peptide (10 μM) [15]. Control – peptide-free magnetic liposomes. *p* < 0.05. The data are mean ± S.E.M. calculated from three independent experiments each performed in triplicate.

To characterize the functional properties of the resultant peptidoliposomes, we used a gp120 core protein that lacks the V1/V2 loop region [13]. Although the coupling between the CD4 and CCR5 binding events is impaired in the gp120 core [29], the corresponding binding epitopes remain intact [13]. Thus, the gp120 core is a suitable target to study the structural determinants of gp120 recognition by the co-receptor. Peptidoliposomes displaying different combinations of CCR5-peptidomimetics were evaluated for their ability to bind soluble gp120 *in vitro*. Each CCR5 epitope, N-terminus or ECL2, promoted soluble monomeric gp120 binding to the peptidoliposomes (Fig 3). As expected for the gp120 core interacting with the co-receptor in a CD4-independent mode [29], pretreatment of gp120 with a soluble CD4-mimetic peptide M48 (sCD4M48: **Tpa**-NLHFCQLRCKSLGLLGRCA**dP**TFCACV, Tpa–Thiopropionyl, dP–(D)-proline) [15] did not significantly enhance gp120 binding to CCR5-peptidoliposomes (Fig 3).

To test whether the CD4- and CCR5-peptidomimetics displayed simultaneously on the surface of the magnetic liposomes would assemble into functional gp120 recognition units, we generated a palmitoylated version of M48:

(3) CD4M48-1PAL:
**Tpa**-NLHFCQLRC-(Lys-PEG8-PAL)-SLGLLGRCA**dP**TFCACV-NH_2_,

The crystal structure of the gp120 complex with a similar CD4-mimetic peptide CD4M33 (PDB: 1YYL) [30] shows that both ends of the peptide are sterically hindered, and as such, their modification, which would affect binding, should be avoided. Therefore, M48 was functionalized with palmitoyl via the side-chain of a core lysine that, based on the structure of the complex, faces the solvent [30]. The ectodomain of CD4 consists of four immunoglobulin-like domains: D1, D2, D3, and D4. The gp120 binding epitope of CD4 is located within D1, the most distant from the cell surface CD4 domain. Conformational flexibility within the CD4 ectodomain conferred by the presence of a hinge region may facilitate virus-cell interactions [31]. Following this logic, M48 was attached to the palmitoyl group via a flexible, hydrophilic polyethylene glycol linker PEG8 (H-(O-CH_2_-CH_2_)_8_-OH).

Soluble gp120 was shown to bind similarly to CD4- or CCR5-peptidoliposomes (Fig 3). Binding was not significantly enhanced in the presence of a combination of CD4- and either CCR5 N-terminus- or ECL2-peptidomimetics. However, binding was strongly enhanced when all the components of the gp120 recognition machinery (CD4; CCR5 N-terminus and ECL2 epitopes) were displayed simultaneously on the peptidoliposome surface (Fig 3). Thus, the peptidomimetics corresponding to different gp120 recognition epitopes exhibited an additive effect on the ability of the peptidoliposomes to bind soluble monomeric gp120.

### Stable analogs of CCR5 N-terminus peptidomimetics

The marked instability of the sulfo-tyrosine groups essential for high affinity CCR5 interaction with Env constitutes a limitation of using CCR5-containing peptidoliposomes in cell-based assays [32, 33]. It has recently been demonstrated that peptidomimetics of the CCR5 N-terminus, whose sulfo-tyrosine residues were replaced by a non-hydrolysable isostere [phenylmethanesulfonate (Phe-CH2-SO_3_^-^, FSN)], maintained their ability to bind gp120 with high-affinity [34]. Thus, we synthesized the CCR5 N-terminus peptidomimetic with three FSN residues at positions 3, 10 and 14:

(4) NT-3FSN-CCR5-PAL:
  MDF-(CH2-SO_3_^-^)QVSSPIF-(CH2-SO_3_^-^)DINF-(CH2-SO_3_^-^)YTSEPAQKINVKQ-Lys-(PAL)-NH_2_.

As shown in Fig 3, the combination of N-terminus 3FSN-CCR5-peptidomimetics with CCR5 ECL2- and CD4-peptidomimetics yielded peptidoliposomes whose soluble gp120 binding efficiency was similar to that of the peptidoliposomes with the sulfo-tyrosine peptidomimetics.

### CCR5-peptidoliposomes inhibit HIV-1 infectivity

**Fig 4:**
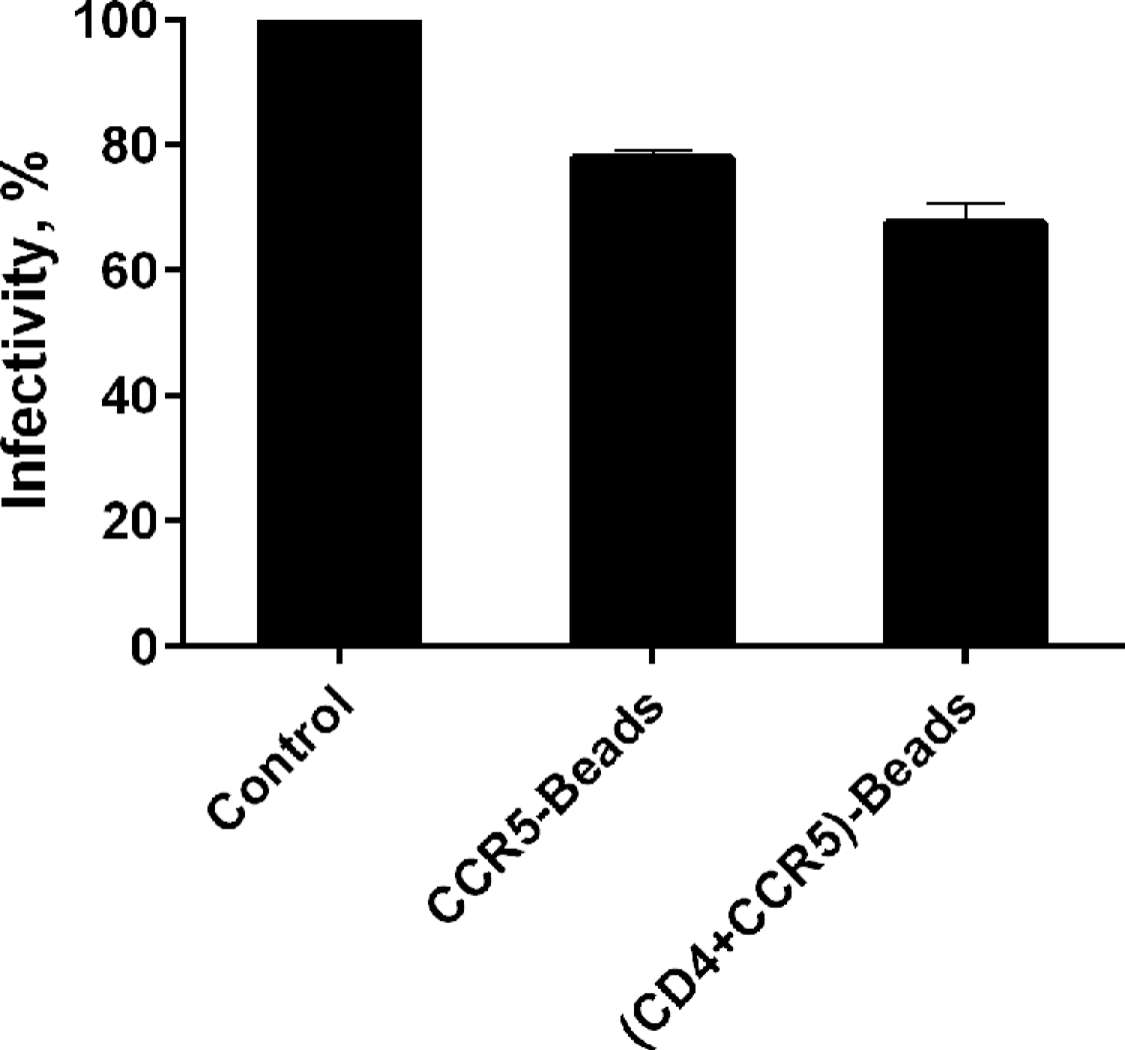
Effect of co-display of CD4- and CCR5-peptidomimetics on the ability of CCR5-peptidoliposomes to inhibit HIV-1. R5-tropic JRFL-pseudotyped HIV-1 was co-incubated for 2 h with: CCR5-Beads–peptidoliposomes containing 5% each of NT-3FSN-CCR5-PAL and ECL2-CCR5-2PAL, or (CD4+CCR5)-Beads–peptidoliposomes containing 5% each of CD4M48-PAL, ECL2-CCR5-2PAL and NT-3FSN-CCR5-PAL. Peptide-free magnetic liposomes were used as control (set to 100% infectivity). The virus was separated from the beads by a magnetic field and TZM-HeLa-β-gal target cells were infected for 4 h. β-gal expression was carried out 48 h post infection. The data are mean ± S.E.M. calculated from three independent experiments each performed in duplicate.

**Fig 5:**
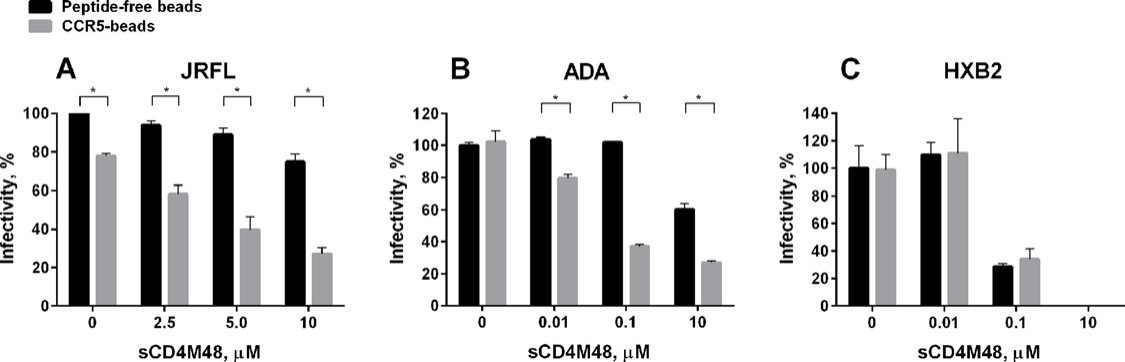
CCR5-peptidoliposomes enhance the ability of soluble CD4 mimetic to inhibit infection of R5-tropic HIV-1. CCR5-peptidoliposomes were co-incubated for 2 h with R5-tropic JRFL-pseudotyped HIV-1 (A); R5-tropic ADA-pseudotyped HIV-1 (B); or X4-tropic HXB2-pseudotyped HIV-1 (C), in the presence or absence of different concentrations of sCD4M48. Peptide-free magnetic liposomes (in the absence of sCD4M48) were used as control (set to 100% infectivity). The virus was separated from the beads by a magnetic field and TZM-HeLa-β-gal target cells were infected for 4 h. β-gal expression was carried out 48 h post infection. An unpaired *t*-test (two-tailed) was used to assess the significance of the difference in the means observed between the two groups indicated, *p* < 0.05. The data are mean ± S.E.M. calculated from three independent experiments each performed in duplicate.

Peptidoliposomes containing different combinations of the CCR5 (N-terminus and ECL2) and CD4-peptidomimetics were initially tested for their ability to inhibit infection of TZM-HeLa-β-gal target cells by R5-tropic JRFL-pseudotyped HIV-1. Pre-incubation of viral particles with the magnetic peptidoliposomes displaying the complete set of CCR5- and CD4-peptidomimetics inhibited viral infectivity by about 30% compared to the control (peptide-free magnetic liposomes) (Fig 4). This was not significantly different, however, from the ability of CCR5-peptidoliposomes alone (in the absence of sCD4M48) to inhibit HIV-1 infection (Fig 4). When different concentrations of sCD4M48 were used instead of the liposome-embedded CD4-mimetics, the treatment with CCR5-peptidoliposomes led to inhibition of viral infectivity at magnitudes proportional to sCD4M48 concentrations (Fig 5A). At the same concentrations, sCD4M48 alone was only marginally effective at inhibiting the infection.

To further evaluate the inhibitory ability and specificity of the CCR5 mimetics, other HIV-1 isolates were tested in the infection assays. Dependence of the inhibition of HIV-1 infectivity by CCR5-peptidoliposomes on sCD4M48 concentration was observed for ADA-pseudotyped R5-tropic virus (Fig 5B). Interestingly, unlike the infectivity of the JRFL isolate, that of the ADA isolate was not affected by the presence of CCR5-peptidoliposomes in the absence of sCD4M48. We also tested the effect of CCR5-peptidoliposomes on the infectivity of X4-tropic (use CXCR4 as co-receptor) HXB2-pseudotyped HIV-1 (Fig 5C). In this case, CCR5-peptidoliposomes did not affect the ability of sCD4M48 to inhibit viral infection, indicating that the structural determinants that contribute to Env co-receptor usage specificity were preserved in the CCR5-mimetics.

**Fig 6:**
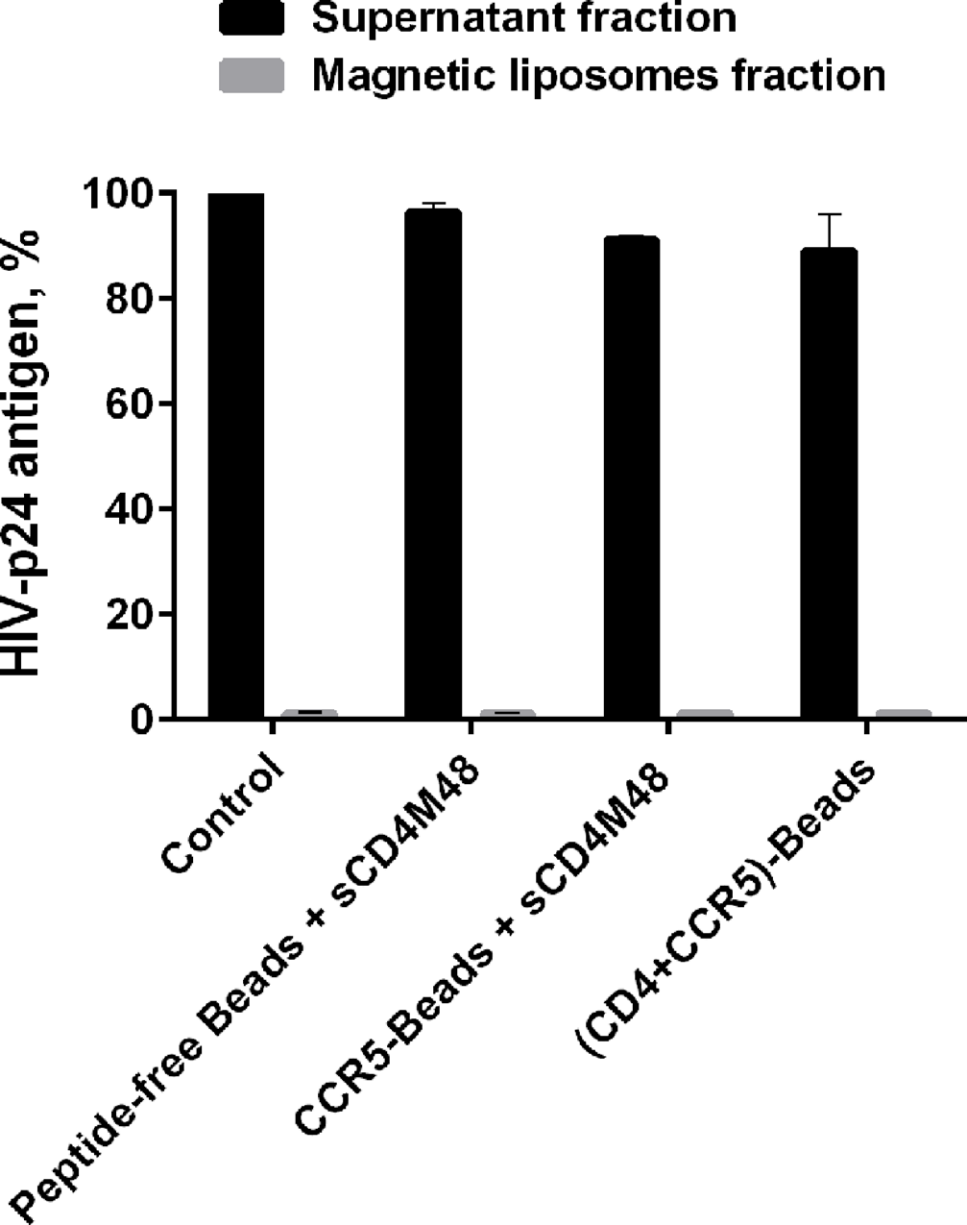
HIV-1 incubation with CCR5-peptidoliposomes does not deplete viral loads. JRFL-pseudotyped HIV-1 was incubated for 2 h with peptidoliposomes in the presence or absence of M48 peptide–sCD4M48 (10 μM). CCR5-Beads - peptidoliposomes containing 5% each of NT-3FSN-CCR5-PAL and ECL2-CCR5-2PAL; (CD4+CCR5)-Beads - peptidoliposomes containing 5% each of CD4M48-PAL, ECL2-CCR5-2PAL and NT-3FSN-CCR5-PAL. At the end of the incubation, the supernatant and liposome fractions were analyzed by ELISA for the presence of HIV-p24 antigen as described in the Materials and Methods. Peptide-free magnetic liposomes were used as the control (p24 count in the supernatant set to 100%). The data are mean ± S.E.M. calculated from three independent experiments each performed in duplicate.

The observed reduction in virus infectivity can be explained by the adherence of particles to the peptidoliposomes, which will cause a decrease in the virus titer following incubation. Alternatively, transient interaction with CCR5-peptidomimetics may accelerate the process of virus Env inactivation triggered by soluble CD4-mimetics. To distinguish between these possibilities, after their incubation with cells, the peptidoliposome and supernatant fractions were evaluated for the presence of HIV-p24 antigen. No significant difference in HIV-p24 antigen levels was observed between the treatments with the control (peptide-free magnetic liposomes) versus that with CCR5-peptidoliposomes. In both cases, essentially the entire viral load was found in the supernatant (Fig 6). We concluded that sCD4M48-primed HIV-1 interacts with CCR5-peptidoliposomes transiently and that such interaction is sufficient to promote virus Env inactivation and a corresponding inhibition of infection.

## Discussion

Key players in HIV-1 infection, CCR5 and CXCR4 GPCRs function as co-receptors for virus entry into CD4^+^ target cells [2]. Host CD4 receptor binding with the viral glycoprotein gp120 induces in the Env a cascade of conformational changes that result in the exposure of a gp120 high-affinity co-receptor binding site. This process culminates in the formation of a gp41 pre-hairpin intermediate that facilitates virus-cell membrane fusion [3]. In energetic terms, CD4-gp120 binding energy is invested in the stabilization of the transition state (lowering the energetic barrier), which effectively separates the inactive state of the gp120-gp41 complex from a more thermodynamically stable, fusion-competent, active state. It appears, however, that the amount of free energy released is insufficient to insure efficient transition at 37°C, and as a result, Env/CD4 is trapped in a “local minimum”, i.e., a high-energy, metastable intermediate that cannot support fusion. In the context of a target cell, subsequent Env binding to the host co-receptor further decreases the energetic barrier and catalyzes gp120-gp41 complex transition to the activated fusion-competent conformation. In the absence of a target cell (when the metastable intermediate is induced by sCD4 binding), uncatalyzed transition may occur spontaneously at a rate that correlates inversely with the depth of the energetic well in which the metastable intermediate is trapped. In the absence of an appropriate fusion target (i.e., cell), the transition ultimately results in the permanent loss of viral infectivity. The fact that some sCD4-primed HIV-1 isolates spontaneously escape the intermediate metastable state at a measurable rate indicates that the amount of free energy required for Env to accomplish the transition is generally low. Differences in the susceptibility of HIV-1 isolates to inhibition by sCD4 have thus been attributed to the varied kinetics of the spontaneous decay of the Env intermediate [2, 9]. Therefore, the clinical use of sCD4 may be limited by slow inactivation kinetics that would allow sCD4-primed virus to infect the co-receptor-expressing cells before the loss of infectivity.

The rationale behind this research was to explore novel approaches to boosting the inhibitory potential of sCD4 mimetics by catalyzing the transition of Env to its inactive state. We hypothesized that exposure of the HIV-1 Env trapped in the metastable state to the co-receptor’s functional epitope outside the target cell would accelerate the transition of Env to the inactive state, thus rendering it inactive before the virus diffuses to within reach of its target cell. Assuming that the co-receptor’s functional epitope is a complex, conformational epitope comprising elements of the extracellular domain that are situated far from each other in the receptor’s primary structure, we suggested that the reconstitution of the functional epitope in its 3D complexity would result in more efficient mimicry of the functional properties of the native co-receptor than would be possible with the isolated peptides derived from co-receptors.

The reconstitution of whole CCR5 and CXCR4 co-receptors in an artificial lipid bilayer, described in previous studies [12, 35–37], yielded molecules whose functional properties were similar to those of native membrane receptors. The process of whole-receptor liposome formation, however, is cumbersome, as it entails the expression and purification of the receptors from the detergent-solubilized cell membranes followed by receptor reconstitution into a lipid bilayer. To simplify the system, we attempted to reconstitute CCR5 gp120-binding epitopes by functionalizing the isolated elements (peptides) of the extracellular domain (ECD), namely, N-terminus and ECL2, with a hydrophobic anchor(s), endowing these elements with the ability to incorporate spontaneously into a lipid bilayer and display efficiently on the surfaces of magnetic beads. This approach both imposes on the ECD elements spatial constraints similar to those found in the native receptors expressed on cells and also maintains their capacity for lateral mobility.

Alike whole-receptor-containing liposomes, the resultant CCR5-peptidoliposomes efficiently bound recombinant soluble gp120 (Fig 3). Interaction with soluble gp120 was significantly enhanced when the peptidoliposomes displayed the entire repertoire of the CD4 and CCR5 gp120-recognition epitopes (Fig 3). Since our soluble recombinant gp120 is a monomeric protein, the effects of avidity on binding can be ruled out, indicating that CCR5- and CD4-peptidomimetics, introduced as separate entities, associate within the lipid bilayer to form functional entities that target individual gp120 molecules. Thus, our results imply that CCR5-peptidoliposomes represent a viable alternative for the whole-receptor-liposomes, and as such, they can be exploited to study the structure-activity relationships of gp120 interaction with its co-receptors. An additional advantage of using the peptidoliposomes instead of whole-receptor-liposomes is that the former approach entails the possibility of dissecting the gp120-binding epitope into its components–namely, N-terminus or ECL2 –which will facilitate studies to elucidate their respective roles in gp120 recognition, including the mechanism of acquired drug resistance.

The use of CCR5-displaying peptidoliposomes alone was ineffective at inhibiting R5-JRFL or ADA HIV-1 infection. However, in the presence of soluble sCD4M48, the inhibition of infectivity of R5-tropic viruses increased as a function of sCD4M48 peptide concentration (Fig 5). Significantly, the use of sCD4M48 alone at the concentrations tested exhibited poor inhibitory effects on HIV-1 infection. In this study, we used M48, a soluble CD4 mimetic peptide whose ability to inhibit JRFL- and ADA-pseudotyped HIV-1 has not been tested [15]. The relatively moderate inhibitory effect demonstrated by CCR5-peptidoliposomes on HIV-1 infectivity (up to 70%) may be explained by a low occupancy of viral gp120 by sCD4M48 due to low apparent affinity of the latter for the viral envelopes that were tested. Nevertheless, the exposure of CD4-primed viruses to CCR5-peptidoliposomes enhanced the extent of virus Env inactivation, suggesting that the resistance of certain HIV-1 isolates to sCD4 inhibition could be circumvented by interaction with CCR5 mimetics.

Although the interaction of CD4-primed HIV-1 with the CCR5 peptidoliposomes appeared to be transient (indicative of low affinity), we suggest that the binding event triggers a process leading to irreversible inactivation of the virus. This is in agreement with the above-mentioned Env activation/inactivation scheme, suggesting that only a small amount of free energy is required to catalyze the escape of Env from its metastable intermediate state, such that even low-affinity interaction of CD4-activated Env with the co-receptor may be sufficient to complete the transition of Env to its activated state, resulting − in the absence of target cells − in permanent virus inactivation. The nature of the permanently inactive state that results from the sCD4-induced conformational transition, however, remains uncertain. It has been proposed that sCD4 disrupts the non-covalent association of the gp120 and gp41 envelope glycoproteins, causing gp120 shedding and rendering virions noninfectious [38–40]. We are currently testing this hypothesis in terms of the CCR5 mimetics described in this study.

The low energy requirement for the activation of CD4-primed Env indicates that the virus may have evolved to utilize only a fraction of the available co-receptor-binding potential, suggesting a potential functional redundancy within various regions of the co-receptor ectodomain. Such redundancy would allow the virus to adjust/expand its co-receptor usage, thereby avoiding the selective-pressure imposed by CCR5-targeted drugs [2, 41].

Our results may indicate a new paradigm in anti-HIV drug development. CCR5-mimetics evolved for high-affinity binding to Env may not necessarily be the most effective or universal inhibitors of virus entry. A directed evolution and selection process aimed at increasing the binding affinity of CCR5 mimetics to Env may result in inhibitors whose action relies solely on competition with the membrane co-receptor without actually inactivating viral Env or, alternatively, which may even stabilize the Env intermediate state. In addition, since such inhibitors were evolved to bind a particular Env isolate, their inactivation profile is expected to be rather narrow, and as such, their inhibition potential would be easily circumvented by drug-resistant mutations. In the case of drugs that target CCR5 directly, the mechanism of acquired resistance involves the adaptation of Env to utilize the drug-bound conformation of CCR5, demonstrating the viral ability to rely on alternative regions of the co-receptor’s ectodomain for fusion and entry [2, 41]. In native co-receptors, free and drug-bound forms (conformations) are fixed, which permits the virus to utilize only one of these conformations more efficiently. We hypothesize that our CCR5-peptidomimetics can function as bait for CD4-primed HIV-1 by displaying a complete repertoire of gp120-recognition epitopes embedded in the artificial lipid membrane, thus closely mimicking CCR5-expressing target cells. In the peptidoliposomes, many of the spatial constraints characteristic of native co-receptors are absent, and the functional unit exists as an assembly of alternating and interchangeable conformations (conformers), all accessible to the virus. Each of these conformers could potentially be stabilized by Env via affinity selection. Therefore, the probability that Env gp120 will evolve into a variant evading interaction with CCR5-peptidoliposomes while maintaining its ability to engage the cellular co-receptors is rather low.

We anticipated that peptidoliposomes containing functional mimetics of both CD4 and CCR5 would have a higher HIV-1 inactivation potential compared to that of CCR5-peptidoliposomes in the presence of soluble sCD4M48 due to the spatial proximity of these recognition epitopes and the corresponding favorable entropic effects on complex formation. However, in the current research, peptidoliposomes displaying both CCR5- and CD4-mimetics exhibited levels of HIV-1 inactivation similar to that of CCR5-peptidoliposomes alone (in the absence of sCD4M48) (Fig 4). The gp120-recognition epitope of the CD4 receptor ectodomain is located within the N-terminal D1, the most remote domain from the cell surface, and it is positioned at a calculated average distance of 100 Å from the cell membrane plane [31]. In the current work, we used PEG-8 as a flexible linker to attach the CD4-peptidomimetic to the palmitoyl group. The PEG-8 moiety positions the CD4 gp120-binding epitope at an average distance of 15 Å from the liposome surface [42], which may explain the limited accessibility of the CD4 gp120-binding epitope in the mimetics to the intact viral particles and the consequently low HIV-1 inactivation efficiency of (CD4+CCR5)-peptidoliposomes. A 15-Å clearance, apparently adequate for binding soluble monomeric gp120, may not be sufficient for productive interaction with intact virus. Therefore, mimetics that exploit longer spacers ([PEG-8]_*n*_, *n* = 2–4) to attach the CD4 moiety to the palmitoyl anchor will be evaluated in future work.

Likewise, related studies by our group are currently evaluating the ability of peptidoliposomes displaying CXCR4 functional epitopes to inhibit HIV infection. Such investigations are of importance, as the use of the CXCR4 co-receptor is correlated with late HIV infection and AIDS. Peptidoliposome-based mimetics that simultaneously display functional epitopes of both CCR5 and CXCR4 may be of particular interest.

Finally, liposome-based mimetics of the cellular Env-recognition machinery may constitute a valuable tool to study the molecular mechanism of co-receptor-induced Env structural transition and to unravel the delicate balance between CD4/CCR5-induced activation versus inactivation of HIV-1 Env. In addition, the use of mimetics may enable the respective contributions of individual co-receptor epitopes to the Env recognition process and to the mechanism of acquired resistance to drugs to be elucidated.

During the preparation of this manuscript, Farzan and colleagues published a study [43] that describes inhibitors of HIV infection comprising CD4 domains 1 and 2 fused to the human IgG-1 Fc domain and to a sulfo-peptide derived from a HIV-1 neutralizing antibody that binds Env in a manner similar to that of CCR5-derived peptides. The CD4-CCR5 fusion mimetics demonstrated improved binding to Env trimers, neutralized a wider panel of HIV-1 isolates, and were less efficient in promoting HIV-1 infection in CCR5^+^/CD4^−^ cells as compared with CD4-mimetics. These findings are in a qualitative agreement with the results of our study describing the functional properties of CCR5-peptidoliposomes. The study by Farzan’s group, however, did not provide a conclusive explanation for the origin of the synergistic effect of the CD4 and CCR5 moieties on the ability of the mimetics to inhibit HIV-1 infection, and suggested that the reduced capacity of the inhibitors to promote HIV-1 infection results from a competition between the CCR5 moiety and the membrane co-receptor for Env binding [43]. Our study, however, indicates that interaction of CD4-primed virus with a CCR5-mimetic may irreversibly strip the virus of its ability to infect cells by catalyzing the transition of Env into a thermodynamically stable, activated state, suggesting that the improved performance of CD4-CCR5 fusion mimetics in inhibiting HIV-1 infection may stem from their ability to irreversibly inactivate the virus.
