# Correction: Functional Mimetics of the HIV-1 CCR5 Co-Receptor Displayed on the Surface of Magnetic Liposomes

**DOI:** 10.1371/journal.pone.0146309

**Published:** 2015-12-29

**Authors:** The PLOS ONE Staff

There is an error in the affiliation and special designation of the seventh author. Dr. Stanislav Engel is a corresponding author and is not affiliated with #4, but with #2 Department of Clinical Biochemistry and Pharmacology, Faculty of Health Sciences, Ben-Gurion University of the Negev, Beer-Sheva, Israel; and #3 National Institute for Biotechnology in the Negev, Beer-Sheva, Israel.

## References

[1] Kuzmina A, Vaknin K, Gdalevsky G, Vyazmensky M, Marks RS, Taube R, et al (2015) Functional Mimetics of the HIV-1 CCR5 Co-Receptor Displayed on the Surface of Magnetic Liposomes. PLoS ONE 10(12): e0144043 doi: 10.1371/journal.pone.0144043 26629902 10.1371/journal.pone.0144043 PMC4667905

